# (*E*)-1-[1-(6-Bromo-2-oxo-2*H*-chromen-3-yl)ethyl­idene]thio­semicarbazide

**DOI:** 10.1107/S1600536810019240

**Published:** 2010-05-29

**Authors:** Afsheen Arshad, Hasnah Osman, Kit Lam Chan, Jia Hao Goh, Hoong-Kun Fun

**Affiliations:** a School of Chemical Sciences, Universiti Sains Malaysia, 11800 USM, Penang, Malaysia; b School of Pharmaceutical Sciences, Universiti Sains Malaysia, 11800 USM, Penang, Malaysia; c X-ray Crystallography Unit, School of Physics, Universiti Sains Malaysia, 11800 USM, Penang, Malaysia

## Abstract

The title compound, C_12_H_10_BrN_3_O_2_S, exists in an *E* configuration with respect to the C=N bond. The approximately planar 2*H*-chromene ring system [maximum deviation = 0.059 (1) Å] is inclined at a dihedral angle of 17.50 (5)° with respect to the mean plane through the thio­semicarbazide unit and an intra­molecular N—H⋯N hydrogen bond generates an *S*(5) ring. In the crystal structure, adjacent mol­ecules are linked by N—H⋯S hydrogen bonds, forming [010] chains built up from *R*_2_^2^(8) loops, such that each S atom accepts two such bonds. These chains are further inter­connected into sheets parallel to the *ab* plane *via* short Br⋯O inter­actions [3.0732 (13) Å] and a π–π aromatic stacking inter­action [3.7870 (8) Å] is also observed.

## Related literature

For general background to and applications of the title thio­semicarbazide compound, see: Anderson *et al.* (2002 ▶); Chulian *et al.* (2009 ▶); Desai *et al.* (1984 ▶); Finn *et al.* (2004 ▶); Hofmanová *et al.* (1998 ▶); Hoult & Payá (1996 ▶); Kimura *et al.* (1985 ▶); Laffitte *et al.* (2002 ▶); Mitscher (2002 ▶); Moffett (1964 ▶); Pillai *et al.* (1999 ▶); Shukla *et al.* (1984 ▶); Tassies *et al.* (2002 ▶); Weber *et al.* (1998 ▶). For the preparation, see: Moamen *et al.* (2009 ▶). For graph-set descriptions of hydrogen-bond ring motifs, see: Bernstein *et al.* (1995 ▶). For a related structure, see: Arshad *et al.* (2010 ▶). For the stability of the temperature controller used for the data collection, see: Cosier & Glazer (1986 ▶).
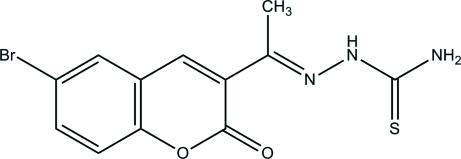


## Experimental

### 

#### Crystal data

C_12_H_10_BrN_3_O_2_S
*M* *_r_* = 340.20
Triclinic, 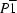

*a* = 6.3796 (6) Å
*b* = 8.1260 (7) Å
*c* = 13.3756 (12) Å
α = 106.697 (2)°
β = 95.095 (2)°
γ = 98.925 (2)°
*V* = 649.57 (10) Å^3^
*Z* = 2
Mo *K*α radiation
μ = 3.33 mm^−1^
*T* = 100 K
0.73 × 0.20 × 0.15 mm

#### Data collection

Bruker APEXII DUO CCD diffractometer
Absorption correction: multi-scan (*SADABS*; Bruker, 2009 ▶) *T*_min_ = 0.196, *T*_max_ = 0.637
19355 measured reflections
5036 independent reflections
4733 reflections with *I* > 2σ(*I*)
*R*_int_ = 0.020

#### Refinement

*R*[*F* ^2^ > 2σ(*F* ^2^)] = 0.021
*wR*(*F* ^2^) = 0.084
*S* = 1.17
5036 reflections
185 parameters
H atoms treated by a mixture of independent and constrained refinement
Δρ_max_ = 0.73 e Å^−3^
Δρ_min_ = −0.56 e Å^−3^

### 

Data collection: *APEX2* (Bruker, 2009 ▶); cell refinement: *SAINT* (Bruker, 2009 ▶); data reduction: *SAINT*; program(s) used to solve structure: *SHELXTL* (Sheldrick, 2008 ▶); program(s) used to refine structure: *SHELXTL*; molecular graphics: *SHELXTL*; software used to prepare material for publication: *SHELXTL* and *PLATON* (Spek, 2009 ▶).

## Supplementary Material

Crystal structure: contains datablocks global, I. DOI: 10.1107/S1600536810019240/hb5461sup1.cif

Structure factors: contains datablocks I. DOI: 10.1107/S1600536810019240/hb5461Isup2.hkl

Additional supplementary materials:  crystallographic information; 3D view; checkCIF report

## Figures and Tables

**Table 1:** Hydrogen-bond geometry (Å, °)

*D*—H⋯*A*	*D*—H	H⋯*A*	*D*⋯*A*	*D*—H⋯*A*
N3—H2*N*3⋯N1	0.82 (3)	2.15 (3)	2.6004 (17)	114 (3)
N2—H1*N*2⋯S1^i^	0.73 (3)	2.70 (3)	3.4094 (13)	165 (3)
N3—H1*N*3⋯S1^ii^	0.81 (2)	2.49 (2)	3.3010 (13)	175.6 (19)

Symmetry codes: (i) 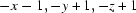; (ii) 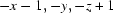.

**Fig. 1.**
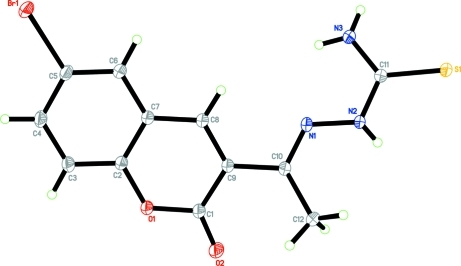
The molecular structure of (I), showing 50 % probability displacement ellipsoids for non-H atoms.

**Fig. 2.**
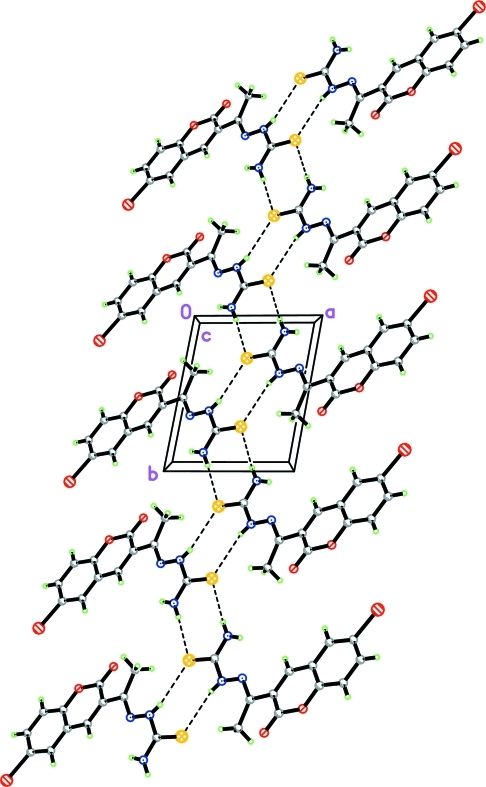
Part of the crystal structure of (I), showing molecules being linked into an infinite chain incorporating zig-zag shaped R22(8) ring motifs along the b axis.

**Fig. 3.**
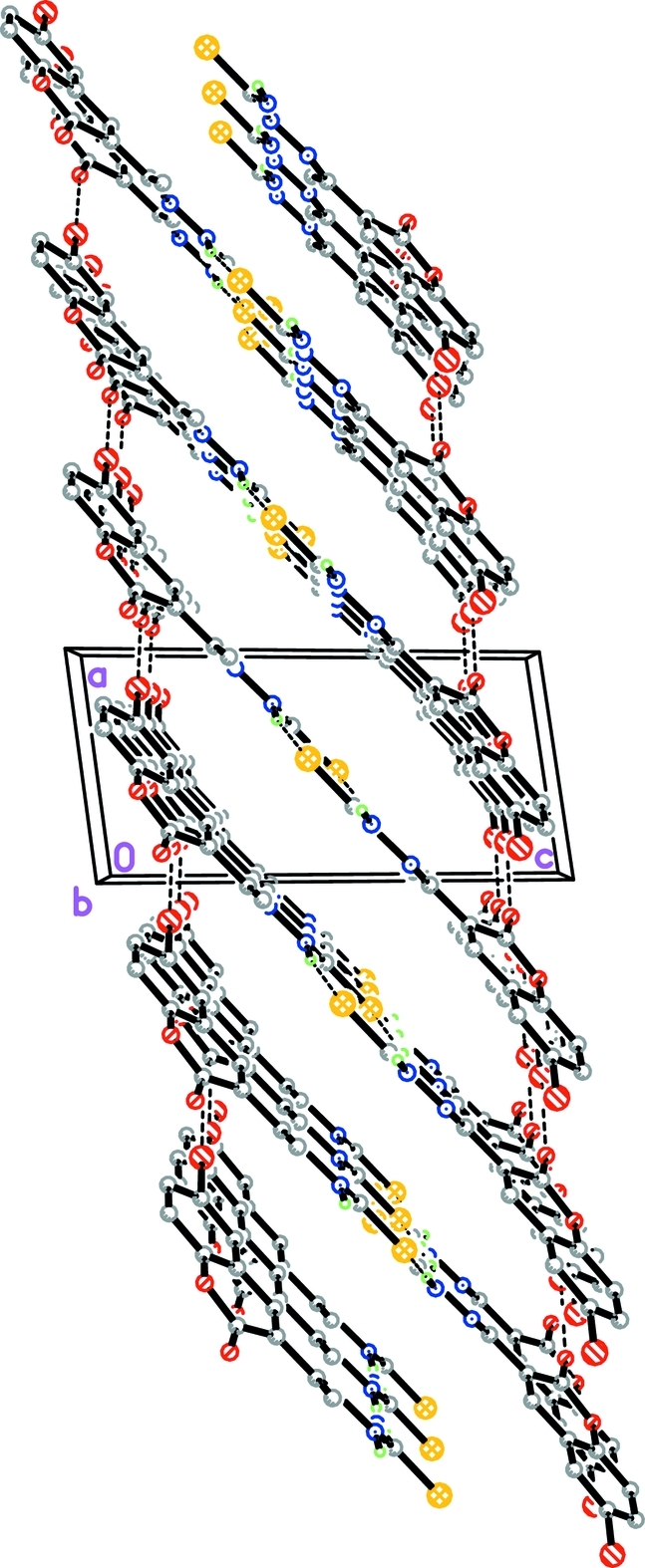
The crystal structure of (I), viewed along the b axis, showing a two-molecule-wide plane parallel to the ab plane. Hydrogen atoms not involved in intermolecular interactions (dashed lines) have been omitted for clarity.

## supplementary crystallographic information

### Comment

Thiosemicarbazide compounds exhibit various biological activities such as anti-bacterial, anti-fungal and especially anti-tuberculosis (Shukla *et al.*, 1984, Desai *et al.*, 1984). Apart from this, coumarins constitute an important class of compounds found throughout the plant kingdom and are known to have diverse activities such as anti-coagulants (Anderson *et al.*, 2002, Tassies *et al.*, 2002), anti-bacterial (Mitscher, 2002, Laffitte *et al.*, 2002), anti-fungal (Moffett, 1964) and cytotoxicity (Weber *et al.*, 1998) properties. The coumarin moiety and related derivatives are also reported to have importance as vasodilators (Hoult & Payá, 1996), anti-mutagenic agents (Pillai *et al.*, 1999), scavengers of reactive oxygen species (Finn *et al.*, 2004), as well as lipoxygenese and cyclooxygenese inhibitors (Kimura *et al.*, 1985, Hofmanová *et al.*, 1998). The title compound exhibits very good anti-bacterial activity against *Escherichia coli* and *Bacillus. subtilus* (Chulian *et al.*, 2009). The objective of this study is to synthesize new derivatives of coumarin-thiosemicarbazide compounds. We present in this paper the crystal structure of this title compound.

The title thiosemicarbazide compound (Fig. 1) exists in a *cis* configuration with respect to the Schiff base C10═N1 bond [N1═C10 = 1.2890 (15) Å; torsion angle C9–C10–N1–N2 = 178.83 (10)°]. The 2*H*-chromene ring system (C1-C9/O1) is approximately planar, with a maximum deviation of 0.059 (1) Å at atom C9. The mean plane through the thiosemicarbazide moiety (N1/N2/C11/N3/S1) forms dihedral angle of 17.50 (5)° with the 2*H*-chromene ring system. Bond lengths and angles are consistent to a closely related structure (Arshad *et al.*, 2010).

In the crystal structure, pairs of intermolecular N2—H1N2···S1 and N3—H1N3···S1 hydrogen bonds (Table 1) form bifurcated acceptor hydrogen bonds which generate two different *R*^2^_2_(8) hydrogen bond ring motifs with zig-zag formation (Fig. 2, Bernstein *et al.*, 1995). These hydrogen bonds link adjacent molecules into two-molecule wide chains along the *b* axis. Intermolecular short Br···O interactions [Br1···O2^iii^ = 3.0732 (13) Å; (iii) x+1, y-1, z] interconnect these chains into two-dimensional planes parallel to the *ab* plane (Fig. 3). The crystal structure is further stabilized by weak *Cg*1···*Cg*1 interactions involving the centroid of the C2-C7 benzene ring [*Cg*1···*Cg*1^iv^ = 3.7870 (8) Å; (iv) -x+1, -y, -z].

### Experimental

Coumarin thiosemicarbazone was prepared by cyclocondensation of 5-bromosalicylaldehyde with ethylacetoacetate and the resulting acetyl coumarin intermediate was then treated with thiosemicarbazide to get the title compound as reported in the literature with some modifications (Moamen *et al.*, 2009). The methanol solution of thiosemicarbazide (5.00 mmol) was added to a solution of 6-bromo-(3-acethylcoumarin) (5.00 mmol) in hot methanol (10 ml) while stirring. The resulting solution was refluxed for 1 h and then pH of the solution was adjusted to 4–5 by adding glacial acetic acid. The solution was again refluxed for 4 h. The title compound was recrystallized from ethyl acetate:ethanol (2:1) to give yellow needles of (I).

### Refinement

H atoms bound to N atoms were located from difference Fourier map and allowed to refine freely [range of N—H = 0.73 (3)–0.82 (3) Å]. All other H atoms were placed in their calculated positions, with C—H = 0.93 or 0.96 Å, and refined using a riding model, with *U*_iso_ = 1.2 or 1.5 *U*_eq_(C). A rotating group model was used for the C12 methyl group.

### Crystal data

**Table:**

C_12_H_10_BrN_3_O_2_S	*Z* = 2
*M**_r_* = 340.20	*F*(000) = 340
Triclinic, *P*1	*D*_x_ = 1.739 Mg m^−^^3^
Hall symbol: -P 1	Mo *K*α radiation, λ = 0.71073 Å
*a* = 6.3796 (6) Å	Cell parameters from 9969 reflections
*b* = 8.1260 (7) Å	θ = 2.7–35.1°
*c* = 13.3756 (12) Å	µ = 3.33 mm^−^^1^
α = 106.697 (2)°	*T* = 100 K
β = 95.095 (2)°	Needle, yellow
γ = 98.925 (2)°	0.73 × 0.20 × 0.15 mm
*V* = 649.57 (10) Å^3^	

### Data collection

**Table:**

Bruker APEXII DUO CCD diffractometer	5036 independent reflections
Radiation source: fine-focus sealed tube	4733 reflections with *I* > 2σ(*I*)
graphite	*R*_int_ = 0.020
φ and ω scans	θ_max_ = 33.5°, θ_min_ = 1.6°
Absorption correction: multi-scan (*SADABS*; Bruker, 2009)	*h* = −9→9
*T*_min_ = 0.196, *T*_max_ = 0.637	*k* = −12→12
19355 measured reflections	*l* = −20→20

### Refinement

**Table:**

Refinement on *F*^2^	Primary atom site location: structure-invariant direct methods
Least-squares matrix: full	Secondary atom site location: difference Fourier map
*R*[*F*^2^ > 2σ(*F*^2^)] = 0.021	Hydrogen site location: inferred from neighbouring sites
*wR*(*F*^2^) = 0.084	H atoms treated by a mixture of independent and constrained refinement
*S* = 1.17	*w* = 1/[σ^2^(*F*_o_^2^) + (0.0569*P*)^2^ + 0.0443*P*] where *P* = (*F*_o_^2^ + 2*F*_c_^2^)/3
5036 reflections	(Δ/σ)_max_ = 0.001
185 parameters	Δρ_max_ = 0.73 e Å^−^^3^
0 restraints	Δρ_min_ = −0.56 e Å^−^^3^

### Special details

**Table:**

Experimental. The crystal was placed in the cold stream of an Oxford Cryosystems Cobra open-flow nitrogen cryostat (Cosier & Glazer, 1986) operating at 100.0 (1)K.
Geometry. All esds (except the esd in the dihedral angle between two l.s. planes) are estimated using the full covariance matrix. The cell esds are taken into account individually in the estimation of esds in distances, angles and torsion angles; correlations between esds in cell parameters are only used when they are defined by crystal symmetry. An approximate (isotropic) treatment of cell esds is used for estimating esds involving l.s. planes.
Refinement. Refinement of F^2^ against ALL reflections. The weighted R-factor wR and goodness of fit S are based on F^2^, conventional R-factors R are based on F, with F set to zero for negative F^2^. The threshold expression of F^2^ > 2sigma(F^2^) is used only for calculating R-factors(gt) etc. and is not relevant to the choice of reflections for refinement. R-factors based on F^2^ are statistically about twice as large as those based on F, and R- factors based on ALL data will be even larger.

### Fractional atomic coordinates and isotropic or equivalent isotropic displacement parameters (Å^2^)

**Table:**

	*x*	*y*	*z*	*U*_iso_*/*U*_eq_	
Br1	0.831903 (19)	−0.130575 (15)	0.134917 (9)	0.01986 (5)	
S1	−0.52737 (6)	0.26734 (4)	0.52668 (3)	0.02208 (7)	
O1	0.39540 (15)	0.46915 (12)	0.12143 (7)	0.01830 (16)	
O2	0.14155 (19)	0.62282 (15)	0.15561 (9)	0.0270 (2)	
N1	−0.08889 (17)	0.33908 (13)	0.34997 (8)	0.01559 (17)	
N2	−0.24110 (18)	0.37175 (14)	0.41584 (8)	0.01611 (17)	
N3	−0.2610 (2)	0.08895 (15)	0.41915 (9)	0.0204 (2)	
C1	0.2259 (2)	0.50976 (16)	0.17462 (10)	0.0177 (2)	
C2	0.48940 (19)	0.32942 (15)	0.12434 (9)	0.01509 (18)	
C3	0.6564 (2)	0.29964 (17)	0.06532 (10)	0.0179 (2)	
H3A	0.7011	0.3720	0.0255	0.022*	
C4	0.75479 (19)	0.15951 (17)	0.06722 (9)	0.0178 (2)	
H4A	0.8675	0.1375	0.0288	0.021*	
C5	0.68424 (19)	0.05161 (16)	0.12688 (9)	0.01630 (19)	
C6	0.51529 (19)	0.07869 (15)	0.18374 (9)	0.01632 (19)	
H6A	0.4670	0.0031	0.2210	0.020*	
C7	0.41777 (18)	0.22285 (15)	0.18420 (9)	0.01446 (18)	
C8	0.25545 (18)	0.27089 (15)	0.24813 (9)	0.01512 (18)	
H8A	0.2113	0.2048	0.2915	0.018*	
C9	0.16407 (18)	0.41112 (15)	0.24698 (9)	0.01454 (18)	
C10	−0.00056 (18)	0.46198 (15)	0.31560 (9)	0.01494 (18)	
C11	−0.33190 (19)	0.23856 (15)	0.44883 (9)	0.01633 (19)	
C12	−0.0508 (2)	0.64307 (16)	0.34349 (10)	0.0196 (2)	
H12A	−0.0674	0.6814	0.4167	0.029*	
H12B	−0.1814	0.6417	0.3013	0.029*	
H12C	0.0641	0.7218	0.3302	0.029*	
H1N2	−0.308 (5)	0.438 (4)	0.420 (3)	0.060 (9)*	
H1N3	−0.317 (3)	0.005 (3)	0.4346 (16)	0.019 (4)*	
H2N3	−0.164 (5)	0.094 (4)	0.383 (3)	0.057 (9)*	

### Atomic displacement parameters (Å^2^)

**Table:**

	*U*^11^	*U*^22^	*U*^33^	*U*^12^	*U*^13^	*U*^23^
Br1	0.02111 (7)	0.01997 (7)	0.02278 (7)	0.01169 (5)	0.00823 (5)	0.00757 (5)
S1	0.02734 (16)	0.01628 (13)	0.03123 (16)	0.01088 (11)	0.02016 (13)	0.01232 (11)
O1	0.0205 (4)	0.0197 (4)	0.0223 (4)	0.0104 (3)	0.0121 (3)	0.0121 (3)
O2	0.0334 (5)	0.0302 (5)	0.0330 (5)	0.0205 (4)	0.0192 (4)	0.0220 (4)
N1	0.0174 (4)	0.0165 (4)	0.0162 (4)	0.0071 (3)	0.0086 (3)	0.0062 (3)
N2	0.0191 (4)	0.0145 (4)	0.0197 (4)	0.0078 (3)	0.0116 (3)	0.0078 (3)
N3	0.0264 (5)	0.0157 (4)	0.0259 (5)	0.0104 (4)	0.0158 (4)	0.0099 (4)
C1	0.0197 (5)	0.0189 (5)	0.0199 (5)	0.0086 (4)	0.0097 (4)	0.0096 (4)
C2	0.0157 (4)	0.0162 (4)	0.0162 (4)	0.0063 (4)	0.0057 (3)	0.0066 (4)
C3	0.0181 (5)	0.0216 (5)	0.0186 (5)	0.0077 (4)	0.0089 (4)	0.0092 (4)
C4	0.0166 (5)	0.0219 (5)	0.0176 (5)	0.0078 (4)	0.0075 (4)	0.0063 (4)
C5	0.0164 (4)	0.0176 (5)	0.0172 (4)	0.0084 (4)	0.0054 (4)	0.0053 (4)
C6	0.0173 (5)	0.0163 (4)	0.0186 (5)	0.0071 (4)	0.0068 (4)	0.0069 (4)
C7	0.0154 (4)	0.0149 (4)	0.0154 (4)	0.0055 (3)	0.0058 (3)	0.0058 (3)
C8	0.0158 (4)	0.0155 (4)	0.0171 (4)	0.0060 (4)	0.0068 (4)	0.0069 (4)
C9	0.0158 (4)	0.0152 (4)	0.0156 (4)	0.0059 (3)	0.0068 (3)	0.0064 (3)
C10	0.0155 (4)	0.0155 (4)	0.0167 (4)	0.0061 (4)	0.0065 (4)	0.0065 (3)
C11	0.0198 (5)	0.0146 (4)	0.0184 (5)	0.0068 (4)	0.0090 (4)	0.0072 (4)
C12	0.0234 (5)	0.0159 (5)	0.0248 (5)	0.0092 (4)	0.0121 (4)	0.0090 (4)

### Geometric parameters (Å, °)

**Table:**

Br1—C5	1.8965 (11)	C3—C4	1.3879 (17)
S1—C11	1.6957 (12)	C3—H3A	0.9300
O1—C2	1.3722 (14)	C4—C5	1.3969 (17)
O1—C1	1.3770 (14)	C4—H4A	0.9300
O2—C1	1.2091 (15)	C5—C6	1.3827 (16)
N1—C10	1.2890 (15)	C6—C7	1.4079 (15)
N1—N2	1.3738 (14)	C6—H6A	0.9300
N2—C11	1.3516 (15)	C7—C8	1.4307 (16)
N2—H1N2	0.73 (3)	C8—C9	1.3613 (15)
N3—C11	1.3288 (15)	C8—H8A	0.9300
N3—H1N3	0.81 (2)	C9—C10	1.4846 (16)
N3—H2N3	0.82 (3)	C10—C12	1.5033 (16)
C1—C9	1.4665 (16)	C12—H12A	0.9600
C2—C3	1.3917 (16)	C12—H12B	0.9600
C2—C7	1.3930 (15)	C12—H12C	0.9600
			
C2—O1—C1	122.75 (9)	C5—C6—H6A	120.6
C10—N1—N2	119.10 (10)	C7—C6—H6A	120.6
C11—N2—N1	117.19 (10)	C2—C7—C6	118.95 (10)
C11—N2—H1N2	111 (3)	C2—C7—C8	118.13 (10)
N1—N2—H1N2	128 (3)	C6—C7—C8	122.83 (10)
C11—N3—H1N3	119.9 (15)	C9—C8—C7	121.52 (10)
C11—N3—H2N3	112 (2)	C9—C8—H8A	119.2
H1N3—N3—H2N3	128 (3)	C7—C8—H8A	119.2
O2—C1—O1	116.08 (11)	C8—C9—C1	119.13 (10)
O2—C1—C9	126.61 (11)	C8—C9—C10	120.96 (10)
O1—C1—C9	117.31 (10)	C1—C9—C10	119.90 (10)
O1—C2—C3	117.34 (10)	N1—C10—C9	113.79 (10)
O1—C2—C7	120.50 (10)	N1—C10—C12	124.38 (10)
C3—C2—C7	122.16 (10)	C9—C10—C12	121.81 (10)
C4—C3—C2	118.48 (11)	N3—C11—N2	117.80 (11)
C4—C3—H3A	120.8	N3—C11—S1	122.45 (9)
C2—C3—H3A	120.8	N2—C11—S1	119.74 (9)
C3—C4—C5	119.89 (10)	C10—C12—H12A	109.5
C3—C4—H4A	120.1	C10—C12—H12B	109.5
C5—C4—H4A	120.1	H12A—C12—H12B	109.5
C6—C5—C4	121.73 (10)	C10—C12—H12C	109.5
C6—C5—Br1	119.11 (9)	H12A—C12—H12C	109.5
C4—C5—Br1	119.11 (9)	H12B—C12—H12C	109.5
C5—C6—C7	118.75 (10)		
			
C10—N1—N2—C11	179.07 (11)	C5—C6—C7—C8	−174.14 (11)
C2—O1—C1—O2	−171.47 (12)	C2—C7—C8—C9	3.45 (17)
C2—O1—C1—C9	7.73 (18)	C6—C7—C8—C9	179.96 (12)
C1—O1—C2—C3	178.59 (11)	C7—C8—C9—C1	3.09 (18)
C1—O1—C2—C7	−1.23 (18)	C7—C8—C9—C10	−178.55 (11)
O1—C2—C3—C4	179.76 (11)	O2—C1—C9—C8	170.56 (14)
C7—C2—C3—C4	−0.43 (19)	O1—C1—C9—C8	−8.54 (18)
C2—C3—C4—C5	0.48 (19)	O2—C1—C9—C10	−7.8 (2)
C3—C4—C5—C6	0.93 (19)	O1—C1—C9—C10	173.08 (11)
C3—C4—C5—Br1	−176.32 (9)	N2—N1—C10—C9	178.83 (10)
C4—C5—C6—C7	−2.35 (18)	N2—N1—C10—C12	0.63 (18)
Br1—C5—C6—C7	174.90 (9)	C8—C9—C10—N1	−18.82 (16)
O1—C2—C7—C6	178.81 (11)	C1—C9—C10—N1	159.52 (11)
C3—C2—C7—C6	−0.99 (18)	C8—C9—C10—C12	159.43 (12)
O1—C2—C7—C8	−4.54 (17)	C1—C9—C10—C12	−22.22 (17)
C3—C2—C7—C8	175.65 (11)	N1—N2—C11—N3	2.93 (17)
C5—C6—C7—C2	2.34 (18)	N1—N2—C11—S1	−177.80 (9)

### Hydrogen-bond geometry (Å, °)

**Table:**

*D*—H···*A*	*D*—H	H···*A*	*D*···*A*	*D*—H···*A*
N3—H2N3···N1	0.82 (3)	2.15 (3)	2.6004 (17)	114 (3)
N2—H1N2···S1^i^	0.73 (3)	2.70 (3)	3.4094 (13)	165 (3)
N3—H1N3···S1^ii^	0.81 (2)	2.49 (2)	3.3010 (13)	175.6 (19)

Symmetry codes: (i) −*x*−1, −*y*+1, −*z*+1; (ii) −*x*−1, −*y*, −*z*+1.
